# The Mechanism Underlying of Long-Term Stable Indigo Reduction State in Indigo Fermentation Using *Sukumo* (Composted *Polygonum tinctorium* Leaves)

**DOI:** 10.3389/fmicb.2021.698674

**Published:** 2021-07-23

**Authors:** Zhihao Tu, Helena de Fátima Silva Lopes, Takashi Narihiro, Isao Yumoto

**Affiliations:** ^1^Bioproduction Research Institute, National Institute of Advanced Industrial Science and Technology (AIST), Sapporo, Japan; ^2^Laboratory of Environmental Microbiology, Graduate School of Agriculture, Hokkaido University, Sapporo, Japan

**Keywords:** *Polygonibacillus*, *Amphibacillus*, *Bacillaceae*, indigo reduction ecosystem, obligate anaerobes, Alkaliphilic bacteria, *Proteinivoraceae*

## Abstract

Indigo fermentation fluid maintains its indigo-reducing state for more than 6 months under open-air. To elucidate the mechanism underlying the sustainability of this indigo reduction state, three indigo fermentation batches with different durations for the indigo reduction state were compared. The three examined batches exhibited different microbiota and consisted of two phases. In the initial phase, oxygen-metabolizing-bacteria derived from *sukumo* established an initial network. With decreasing redox potential (ORP), the initial bacterial community was replaced by obligate anaerobes (mainly *Proteinivoraceae*; phase 1). Approximately 1 month after the beginning of fermentation, the predominating obligate anaerobes were decreased, and *Amphibacillus* and *Polygonibacillus*, which can decompose macromolecules derived from wheat bran, were predominantly observed, and the transition of microbiota became slow (phase 2). Considering the substrate utilization ability of the dominated bacterial taxa, the transitional change from phase 1 to phase 2 suggests that this changed from the bacterial flora that utilizes substrates derived from *sukumo*, including intrinsic substrates in *sukumo* and weakened or dead bacterial cells derived from early events (heat and alkaline treatment and reduction of ORP) to that of wheat bran-utilizers. This succession was directly related to the change in the major substrate sustaining the corresponding community and the turning point was approximately 1 month after the start of fermentation. As a result, we understand that the role of *sukumo* includes changes in the microbial flora immediately after the start of fermentation, which has an important function in the start-up phase of fermentation, whereas the ecosystem comprised of the microbiota utilizing wheat bran underpins the subsequent long-term indigo reduction.

## Introduction

Indigo is one of the oldest dyes used by humans, with evidence of its use dating back 6,000 years (Splitstoser et al., 2016). The vat dye, insoluble indigo, needs to be reduced to its water-soluble form, leuco-indigo, to be used as a fabric dye. Among the traditional process, indigo reduction occurs via natural fermentation, which has been used for European couched woad derived from *Isatis tinctoria* (Clark et al., 1993; Padden et al., 1999; Osimani et al., 2012; Hartl et al., 2015; Milanović et al., 2017) and Japanese *sukumo* (Aino et al., 2010, 2018; Okamoto et al., 2017) and Chinese Landian Yao (Li et al., 2019) derived from *Polygonum tinctorium*. Among the currently used industrial processes, the chemical reductant, sodium dithionite is the preferred reducing agent owing to its high efficiency (Etters, 1989); however, the process is not environmentally friendly. The traditional fermentation-reduction method would be a candidate for environmentally friendly procedures. To make the already established methods more convenient and reliable, it is necessary to understand the fundamentals of indigo fermentation fluids. Several indigo-reducing microorganisms have been identified using culture-based approaches, and their population dynamics has been analyzed using culture-independent molecular approaches as described below.

The indigo-reducing bacterium, *Clostridium isatidis*, has been isolated from a couched woad vat prepared using a traditional medieval European procedure (Padden et al., 1998, 1999). It has also been detected in various woad vats in the United Kingdom and Nigeria as well as cultures grown from viable spores preserved in the 1,000-year-old debris of an Anglo-Scandinavian (Viking) woad vat (Padden et al., 2000). A study analyzing bacterial diversity in fermenting dye vats with woad prepared and maintained in a functional state for approximately 12 months reported that known indigo-reducing bacteria constituted only a small fraction of the unique microcosm (Milanović et al., 2017). The microbiota of couched woad fermentation may be different from that using *sukumo* due to the differences in fermentation condition and in phytochemical constituents between *I. tinctoria* and *P*. *tinctorium*.

The most common source of indigo in the traditional Japanese procedure is *sukumo*, which is composted leaves of *P*. *tinctorium*. Approximately three tons of dried leaves of *P*. *tinctorium* are mixed with the same amount of water and are stacked to a 1-m pile on an earthen floor. Once every 2 to 3 days, the pile of leaves is mixed up and down and an appropriate amount of water to keep the temperature around 60°C. During this process, indican which is the precursor of indigo, is oxidized to indigo and the plant tissue is decomposed. This process takes up to 100 days with appropriate managements using sophisticated techniques by trained craftsperson (Aino et al., 2010; Nakagawa et al., 2021).

Indigo reduction in *sukumo* is achieved by natural fermentation in an extremely alkaline fluid (pH ≥ 10; Aino et al., 2018). At least 11 indigo-reducing species have been isolated from Japanese indigo fermentation fluids (Aino et al., 2018). Aino et al. (2010) investigated the bacterial community associated with the initiation of indigo fermentation by clone analysis and observed that the predominant *Halomonas* spp. (54%) were replaced by indigo reducing *Amphibacillus* spp. (35%) and *Alkalibacterium* (18%) at the beginning of indigo reduction. The mechanism by which the reduction of indigo is initiated has not been elucidated, but it is very important to reliably initiate this process through fermentation. Recently, studies have been performed to promote the reduction of indigo by adding exogenous microorganisms (Jung et al., 2020; Son et al., 2020). Okamoto et al. (2017) examined the microbiota in one early-phase batch and two-aged batches of Japanese indigo fermentation fluid. Although the function of obligate anaerobes is unknown, it was considered that indigo-reducing bacteria are the main components of the bacterial community. The abundance of the indigo-reducing bacteria fluctuated, and each indigo-reducing taxon was replaced by the other indigo-reducing taxa as the fermentation period increased. The transition of bacterial flora in the aged fermentation fluid was slower than that in the early-phase fluid (Okamoto et al., 2017).

Changes in the bacterial community during the early phase in *sukumo* fermentation fluid have previously been evaluated using next-generation sequencing (NGS; Tu et al., 2019a). The indigo-reduction state was achieved after the consumption of oxygen by aerobic bacteria (until day 5); this was followed by an increase in the abundance of the obligate anaerobe *Anaerobranca* and the aerotolerant indigo-reducing *Amphibacillus* (until day 7). The study indicated that all the predominant bacteria that appeared during fermentation were derived from *sukumo*. To understand the mechanisms underlying the sustainability of indigo fermentation fluid, the microbiota of two batches of fermentation fluids with different durations for the indigo-reducing state were analyzed (Tu et al., 2019b). The study showed that early predominance of obligate anaerobes, followed by successive changes into a stable microbiota is important for establishment of a fermentation fluid with long-lasting indigo-reducing state.

In the indigo-reducing state, the fermentation fluid is particularly robust, enabling frequent utilization of the fluid for staining. Although the immersion of fabrics into the fluid provides many opportunities for the introduction of many microbial contaminants and oxygen, the indigo-reducing state lasts more than 6 months. The microbial characteristics responsible for the robustness of this indigo-reduction state in indigo fermentation fluid remain unclear. Functional resiliencies mediated by microbiota ecosystems owing to functional redundancies, have been reported in biofilm and intestinal systems (Luan et al., 2020; Tannock, 2021; Zhao et al., 2021). Here, three batches of indigo fermentation fluids based on Japanese *sukumo* with different indigo-reducing lifetimes and maintenance procedures were evaluated to elucidate the mechanism underlying the transitional changes in microbiota responsible for the formation of the long-lasting indigo-reducing state. The successive changes in bacterial flora in concomitance with diversity changes and microbial interaction networks were analyzed based on statistical correlations among microbial community assemblages.

## Materials and Methods

### Preparation of Indigo Fermentation Fluids

Three batches of *sukumo*-based fermentation fluids were used (Table 1). For all three batches, the high quality *sukumo* produced by a craftsman (A.S.) in Tokushima Prefecture, Japan, was used. For Batches 1 and 2, we added 380 g *sukumo* and the same weight of wood ash (fine powder of burned charcoal prepared from *Quercus phillyraeoides*) to 5 L water. Firstly, the wood ash was added to 5 L water, which was then boiled by simmering on a stove for 10 min. When the wood ash suspension cooled to 60°C at room temperature (approximately 25°C), *sukumo* was added to the supernatant fluid and mixed well. The mixture was then placed at 26°C in a thermostatic room protected from light. The next day, Batch 2 was heated again to 60°C with constant stirring and then returned to the thermostatic room. Batch 1 was not heated the next day. Batch 3 was prepared from 532 g *sukumo* and 7 L wood ash extract by heating to 70°C. It was then placed in the same thermostatic room, heated again to 60°C or 70°C on the next day, and then maintained at 26°C. During the subsequent long-term fermentation, the fluids were stirred every day. The pH of the fermentation fluid was maintained between 10.3 and 11 using the following: Ca(OH)_2_ in addition to Na_2_CO_3_ (2 g), NaOH (500 μL) and lactic acid (500 μL) for pH reduction for Batch 2; Ca(OH)_2_ and of K_2_CO_3_ also were used for initial pH adjustment for Batch 3; and only Ca(OH)_2_ for Batch 1. The pH and the redox potential (ORP) of the fermentation fluids were measured using a pH meter D-71 with a 9625-10D electrode (Horiba, Kyoto, Japan) and a pH/ORP/DO meter D-75 with a 9300-10D electrode (Horiba, Kyoto, Japan), respectively. Wheat bran was added regularly in Batch 1, whereas for Batch 3, the addition was judged by observing the state of the fermented liquor. The differences in the preparation and maintenance procedure of three batches were summarized in Table 1.

**TABLE 1:** Preparation and maintenance procedures for three batches of Japanese indigo fermentation fluids.

Batch No.	*Sukumo* (g)/Wood ash extract (L) content	Pre-heating	First feeding/Frequency	Feeding substance	pH-maintaining substance	Stirring tool (top width)
1	380/5 (76 g/L)	day 0, 60°C	day 35/ every 1–2 weeks	wheat bran (main), wheat gluten, syrup	Ca(OH)_2_	lab spoon (30 mm)
2	380/5 (76 g/L)	day 0, 60°C, day 1, 60°C	No	–	Ca(OH)_2_, Na_2_CO_3_, NaOH	glass rod (5 mm)
3	532/7 (76 g/L)	day 0, 70°C, day 1, 60°C	day 22/ every 2–6 weeks	wheat bran (main), wheat gluten, syrup	Ca(OH)_2_, K_2_CO_3_	lab spoon (30 mm)

### Dyeing Intensity Assessment

The indigo-reduction stage was evaluated by dyeing cotton cloth. During this procedure, a piece of white cotton cloth (∼2 cm × 3 cm) was soaked in the fermentation fluid for 30 s, removed, and exposed to air for oxidation for 5 min. Impurities were rinsed out in flowing tap water. The staining intensity of each fermentation stage was evaluated on the basis of the color of the dyed cloth (Supplementary Figure 2). Batch 2 was fermented for 51 days without feeding, Batch 3 was fermented for 342 days with low-frequency feeding (mainly wheat bran, every 2–6 weeks, with the first feeding on the 22nd day), and Batch 1 was fermented for 364 days with higher frequency feeding (mainly wheat bran, every 1–2 weeks, with the first feeding on the 35th day; Table 1).

### Illumina Sequencing

The samples were centrifuged at 15,000 × *g* for 10 min to obtain the sample pellet. Total bacterial DNA was directly extracted from the sample pellets using ISOIL (Nippon Gene, Tokyo, Japan) according to the manufacturer’s instructions. The V3-V4 region of the bacterial 16S rRNA gene was polymerase chain reaction (PCR)–amplified using the composite pair of primers 341F (5′-CCTACGGGNGGCWGCAG-3′) and 805R (5′-GACTACHVGGGTATCTAATCC-3′). The PCR for samples from Batches 2 and 3 was performed in a 100 μL solution containing 20 μL 5 × Phusion HF buffer (ThermoFisher Scientific, Waltham, MA, United States), 2 mL of 2.5 mM dNTP mixture (TaKaRa, Otsu, Japan), 25 ng of the DNA isolated, 2 U Phusion Hot Start II DNA polymerase (ThermoFisher Scientific), and 50 pmol of each primer. The amplification reactions were performed under the following conditions: initial thermal denaturation at 98°C for 30 s, followed by 25 cycles each of heat denaturation at 98°C for 10 s, annealing at 55°C for 20 s, and extension at 72°C for 30 s. The PCR for Batch 1 samples was performed in a 20 μL solution containing 2 μL 10 × Ex buffer, 1.6 μL of 2.5 mM dNTP mixture, 1 U Ex-Taq (TaKaRa), 1 ng of the DNA isolated, and 1 μL of each 10 μM primer. The amplification reactions were performed under the following conditions: initial thermal denaturation at 94°C for 2 min, followed by 20 cycles each of heat denaturation at 94°C for 30 s, annealing at 55°C for 30 s, and extension at 72°C for 30 s.

For NGS analyses, the first-PCR products for samples were submitted to Hokkaido System Science Co., Ltd. (Sapporo, Japan) or Bioengineering Lab. Co., Ltd. (Sagamihara, Japan). The second PCR was performed with an index-adapted primer to generate paired-end libraries (2 × 301 bp) for NGS performed using the MiSeq platform (Illumina, San Diego, CA, United States).

### NGS Data Analyses

The adapters from raw sequence were cut off using Cutadapt version 1.18. Bioinformatics analysis and output data annotation were performed using QIIME 2 version 2019.10 (Bolyen et al., 2019). Quality control and operational taxonomic unit (OUT: referred as lowest level of identified unit; Edgar, 2017) table construction were executed with the Divisive Amplicon Denoising Algorithm (DADA2; Callahan, 2016), wherein sequences with quality scores ≥30 were selected and OTU grouping was based on 100% similarity. Chimera checking was performed on a pair of FeatureTable and FeatureData artifacts in QIIME 2. The Silva_132_release from the Silva database (Quast et al., 2013; Yilmaz et al., 2014) was used to train the feature classifier for 16S rRNA taxonomic annotation based on the primer pair 341F-805R. Considering that this database is not live-updated, the taxonomic annotation of representative sequences was also blasted against the 16S rRNA database in NCBI^1^ (Miura et al., 2019). Rarefaction curves of observed OTUs and the Shannon index of diversity were plotted based on the QIIME 2 alpha diversity. Jaccard distance principal coordinate analyses (PCoA) and unweighted UniFrac distance PCoA analysis were plotted with based on QIIME 2 beta diversity. EMPEROR software was used to visualize the PCoA plots (Vázquez-Baeza et al., 2013). Alpha and beta diversities were computed at a sampling depth of 6,000 to cover all samples (Lundin et al., 2012). A heatmap was generated based on the OTUs with the QIIME 2 heat map script.

On combining the taxonomic annotation results from QIIME 2 and BLAST, the classifications whose percentage was less than 2% in any sample were removed. The change trend of relative content with fermentation time was subjected to correlation analysis to evaluate the relationships of the main bacterial communities using the programs Psych and Reshape2 in R version 4.0.2. Spearman’s rank correlation coefficient was used to detect correlated bacterial taxa from each fermentation batch in the fermentation process. Cytoscape software version 3.8.0 (Shannon et al., 2013) was used to visualize molecular ecological networks. Predictive functions of the metagenomes of Batch 1 samples were estimated using PICRUSt2 (Douglas et al., 2020). KEGG pathway mapping was assigned in searching in the KEGG database according to the output results.

## Results

### Bacterial Community Analysis on the Basis of NGS and Indigo Reduction

There were 702,293, 142,580, and 137,114 raw sequences obtained from Batch 1 (17 samples), 2 (10 samples including 1 *sukumo* sample) and 3 (10 samples), respectively. After adapter trimming, quality filtering, and chimeric exclusion, 461,620, 95,679, and 104,019 sequences remained, including 13,377 sequences from the *sukumo* sample. Moreover, in each sample of Batch 1, 2, and 3, the lowest available sequences were 19,283, 6,531, and 7,867, respectively.

**FIGURE 1:**
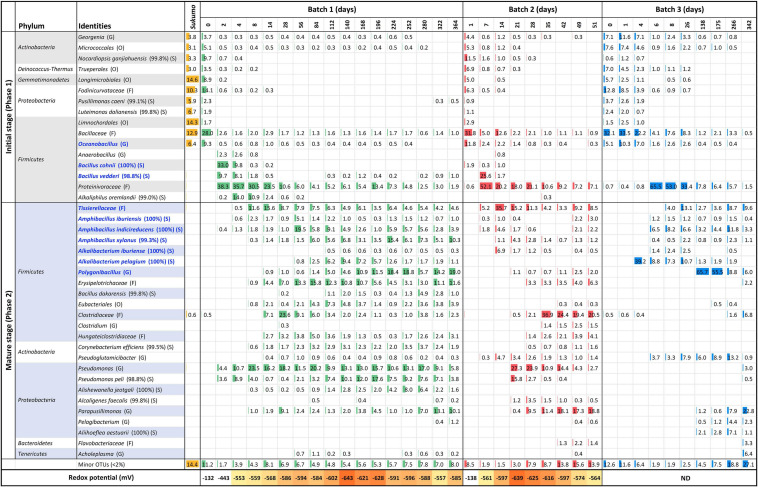
Changes in the relative abundance of bacterial communities (≥2% in any sample) and redox potential, depending on the fermentation age in indigo fermentation fluid Batch 1, 2, and 3, based on 16S rRNA analysis. Blue letters indicate the confirmed indigo-reducing taxa (including unpublished results). The percentages in the brackets indicate the similarities with the known species in the database. Classification hierarchy depends on database similarity as follows: O, order level (<90% similarity); F, family level (≥90, <95%); G, genus level (≥95, <98.6%); and S, species level (≥98.6). ND, no data.

The bacterial community structure of the three batches of *sukumo* fermentation fluid and redox potential was shown in Figure 1. Ranges of weekly pH changes and results of dyeing using fermentation fluids from Batches 1–3 during each incubation period are shown in Supplementary Figures 1, 2, respectively. In addition, the three batches exhibited different life spans of the indigo-reducing state; however, these transitions in microbiota composition consisted of two phases. The features of each phase were as follows: Phase 1 included initial bacterial flora resulting from hot wood ash extract treatment (60–70°C, pH 10.8–11.5). This initial microbiota exhibited a transitional change to that dominated by obligate anaerobes due to a decrease in the redox potential. Phase 2 was a stable state wherein the rate of change in the microbiota was slower than that in the previous phase. This phase ultimately moved toward a deterioration of the indigo-reducing state due to increase in microbiota diversity.

*Sukumo* was pre-treated with hot (60–70°C) ash extract (ca. pH 11) during the preparation of the fermentation fluid. This process resulted in the selection of a heat- and alkali-resistant population that mainly comprised *Bacillaceae* and *Actinobacteria*. Batches 2 and 3 received heat treatment twice (days 0 and 1), and their bacterial constituents were much simpler than those of Batch 1, which received heat treatment only once (Table 1). Concomitant with the oxygen consumption by the initially oxygen-metabolizable bacteria, *Proteinivoraceae* increased until day 2 (0 → 38.3%) in Batch 1. Under the predominance of *Proteinivoraceae* starting from day 2, *Anaerobacillus* sp., *Bacillus cohnii* (reported indigo-reducing bacteria), and *Bacillus vedderi*-like-bacteria (from days 2 to 4), followed by *Alkaliphilus oremlandii*-like bacterium (*Alkaliphilus* sp.), appeared (from days 4 to 8). However, these taxa tended to decrease from days 8 to 14; the obligate anaerobe *Tissierellaceae* increased and an obvious indigo reduction occurred (day 8). We have isolated *Alkalibacterium pelagium* and *B. vedderi*-like bacterium, and *Tissierellaceae*, and confirmed them to be indigo-reducing bacteria (unpublished result). Therefore, indigo reduction did not initiate just by the appearance of indigo-reducing bacteria in Batches 1 and 3 (day 2 in Batch 1 and day 4 in Batch 3).

**FIGURE 2:**
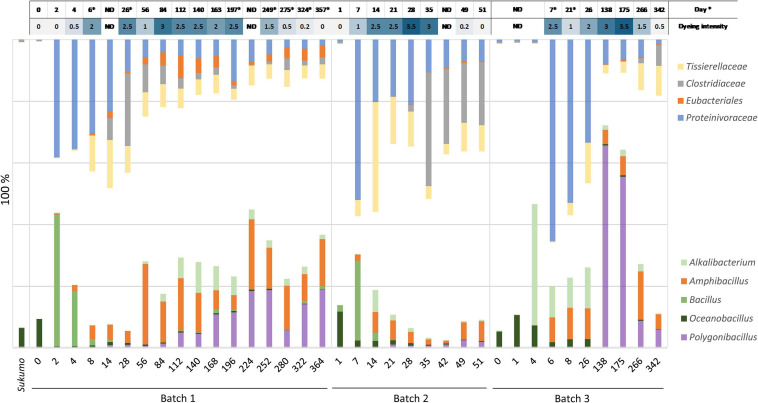
Relationship between dyeing intensity and abundance ratio changes in known facultative anaerobic or aerotolerant indigo-reducing bacteria (lower side), and in obligate anaerobes (upper side) including the indigo-reducing *Tissierellaceae*. ND: no data; * The dyeing date was different from the sampling date for analysis of bacterial community.

The period from days 6–8 to approximately days 26–35 can be considered as the transitional period of the mature phase. After the dominance of *Proteinivoraceae* and *Tissierellaceae* decreased, *Amphibacillus* (from day 56 in Batch 1) and *Polygonibacillus* (from day 84 in Batch 1; from day 138 in Batch 3) increased in Batches 1 and 3. Indeed, the inter-batch differences suggested that the variation in the succeeding microbiota was dependent on the initial treatments and maintenance protocols. During this period, the abundances of the known facultative and aerotolerant anaerobic indigo-reducing taxa decreased drastically in Batches 1 and 2 (Figure 2). After this period, the stable indigo-reduction state lasted for a long period in Batches 1 and 3. However, the lifetime in Batch 2 was short owing to the length of phase 2.

During the late period of the matured phase 2, *Parapusillimonas granuli*-like bacterium, which cannot grow at high pH (e.g., pH 10) and *Erysipelotrichaceae* tended to increase in all the batches. In Batches 1 and 2, *Pseudomonas* was consistently present throughout the fermentation period. However, our finding indicated that *Pseudomonas* spp. did not have a negative effect on the indigo-reducing state because the staining intensities in the *Pseudomonas* spp.-predominant periods were not low (e.g., day 84 in Batch 1 and day 21 in Batch 2). The staining intensity was low on days 280 (dyeing result day 275 presented in Supplementary Figure 2), 49 and 342 in Batches 1, 2, and 3, respectively.

The bacterial community was also visualized using a heat map based on the OTUs (Supplementary Figure 3). The gene sequence corresponding to the OTU described in the output was assigned according to a search of the BLAST database. Batch 1 exhibited five separate clusters, except on day 0. The first cluster mainly consisted of *Proteinivoraceae* and *B*. *cohnii*. This was thought to represent an intermediate state in the change from aerobic metabolism to anaerobic metabolism. The second cluster was characterized by the dominance of *Proteinivoraceae*, *Tissierellaceae*, and *Pseudomonas* (days 8 and 14). In addition to the major members in the second cluster, *Clostridiaceae* and *Erysipelotrichaceae* appeared in the third cluster (days 28 and 58). The most predominant taxon was *Am*. *indicireducens* at day 28, while the most majoring constituent was *Clostridiaceae* at Day 28. Hence, it was thought that this cluster represent an intermediate state of transfer from phase 1 to phase 2. The fourth cluster was from day 84 to 280. This cluster consisted of various taxa and the most major predominant were *Polygonibacillus* and *Am*. *indicireducens*. The last cluster involved days 322 and 364. This was characterized by the absence of *Proteinivoraceae* and the predominance of *Parapusillimonas*. Although Batch 3 samples exhibited similarities with those of Batch 2 samples in the early phase (until day 28), each batch exhibited independent clustering in the later periods of each fermentation.

### Reported Indigo-Reducing Bacterial Community Versus Obligate Anaerobic Community

The relational changes reporting indigo-reducing *Bacillaceae* plus *Alkalibacterium* (oxygen-metabolizing facultative anaerobes plus aerotolerant anaerobes) versus obligate anaerobic *Eubacteriales* (*Clostridiaceae* and *Proteinivoraceae*) plus *Tissierellaceae* are shown in Figure 2. The abundance of *Proteinivoraceae* plus *Tissierellaceae* in Batches 2 and 3 was higher than that in Batch 1. This indicates that the twice heat treatment in Batches 2 and 3 promoted *Proteinivoraceae* plus *Tissierellaceae* propagation. However, the abundance of *Proteinivoraceae* plus *Tissierellaceae* in Batch 2 decreased at day 35. There was at least a 2–18-day gap between the increase or decrease of the abundance reported indigo-reducing species including *Bacillaceae* plus *Alkalibacterium*, and staining intensity (Supplementary Figure 2). This gap was probably due to the fact that the staining intensity was also affected by factors other than the abundance of the known indigo-reducing species. Such other factors may include concentrations of substrates and electron mediators (e.g., flavin). The maximum total abundance of *Bacillaceae* plus *Alkalibacterium* in Batch 2 was 30.5% (day 7) and was typically less than 10% after day 21. The maximum abundance in Batches 1 and 3 were 44.9% (day 224) and 72.2% (day 138), respectively, and typically more than 20% (Figure 2). Thus, the total abundance of *Bacillaceae* plus *Alkalibacterium* in the long-lasting Batches 1 and 3 was considerably higher than that in the short-lasting Batch 2. Among all the indigo-reducing species, including *Bacillaceae* plus *Alkalibacterium*, only *Am*. *indicireducens* showed an increase in the initial indigo reduction or initial drop in ORP in all batches, that is, from 0.39 to 1.31% (day 2 → day 4) in Batch 1, 0.17 to 1.78% (day 1 → day 7) in Batch 2, and 0.23 to 6.49% (day 4 → day 6) in Batch 3. These changes coincided with the change in dominance from *Bacillaceae* to *Proteinivoraceae*. Although the percentages were constantly changing, only *Am*. *indicireducens* was present almost throughout the process in all three batches among known indigo-reducing species. Among the obligate anaerobes, the ratio of *Proteinivoraceae* may be important for the maintenance of the indigo-reducing state. The abundance decreased with the fermentation period.

### Bacterial Diversity Changes

**FIGURE 3:**
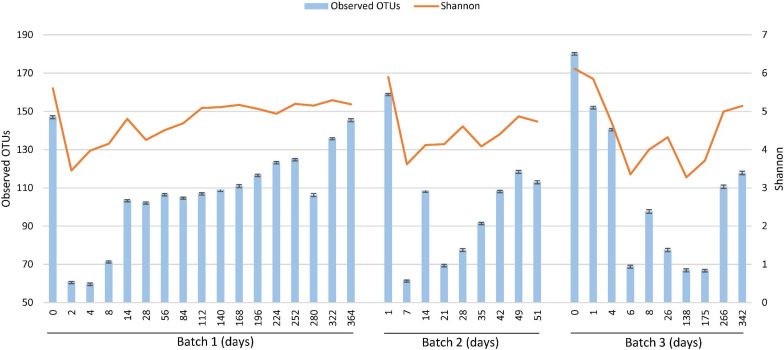
Changes in alpha diversity [observed operational taxonomic units (OTUs) and Shannon index of diversity (Shannon, 1948)] depending on the fermentation period, analyzed using the Divisive Amplicon Denoising Algorithm (DADA2) based on the fermentation period. The 16S rRNA sequencing depth is 6,000 for each sample. The number corresponding to the depth was according to the read number for the coverage of all samples.

Changes in alpha diversity from the rarefaction curves at a sampling depth of 6,000, based on the observed OTUs and Shannon index of diversity, are shown in Figure 3. The bacterial diversity of Batch 3 gradually decreased after the beginning of fermentation (from days 0 to 6) and reached a minimum at the beginning of the reduction. The decrease in diversity may be reflected by the death of bacteria, which originated from the *sukumo*, and was caused by a gradual decrease in the redox potential. A sudden decrease in diversity was observed in Batch 1 on day 2. This finding suggests that both facultative and obligate anaerobes facilitated the elimination of *sukumo*-derived oxygen- metabolizing populations. In Batch 2, although there are no data for days 2–6, the diversity first decreased on day 7 (observed OTU and Shannon index), following which it increased until day 14 (observed OTUs) and then decreased on day 21 (observed OTUs) and day 35 (Shannon index). These decreases in diversity around days 28–35 may be caused by the transitional phase change from *Proteinivoraceae* and *Tissierellaceae* to *Clostridiaceae* in Batch 2. The first change was attributed to the decrease in *sukumo*-derived heat-tolerant aerobes due to substitution by anaerobes adaptable to an environment with low redox potential. The second environmental change may be caused by depletion of initial nutrients derived from *sukumo* at days 21 in Batch 2 (observed OTUs). The decrease in the diversity probably attributed by the depletion of nutrients was also observed in the Shannon index at day 28 in Batch 1 and in the observed OTUs at day 26 in Batch 3 (Figure 3). In the late stage of the mature phase 2, an increasing number of suppressed neutralophilic bacterial populations tended to gradually increase in diversity with an increase in debris in the neutralophilic microenvironment at the bottom of the fermentation fluid.

### Principal Co-Ordinate Analysis

**FIGURE 4:**
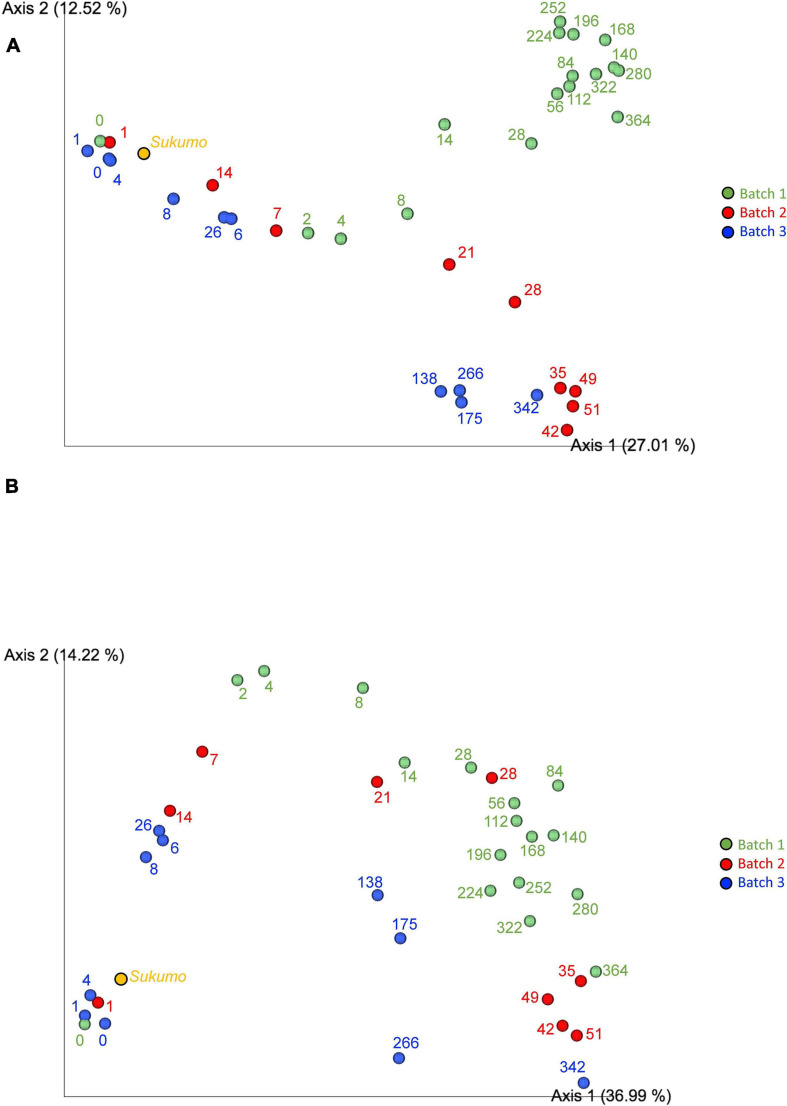
Jaccard distance principal coordinate analysis [PCoA; Fuxman Bass et al., 2013; **(A)**] and unweighted UniFrac distance PCoA analysis [Lozupone and Knight, 2005; **(B)**]. Batch 1, green circles; Batch 2, red circles; and Batch 3, blue circles. Numbers indicate the fermented days. The 16S rRNA sequencing depth is 6,000 for each sample.

Principal coordinate analysis (PCoA) plots based on Jaccard (Figure 4A) and unweighted UniFrac distances (Figure 4B) illustrated similarities and distances of the microbiota among the three batches. It is considered that the common change from the start of fermentation to the end of fermentation between batches indicated that the changes in the *sukumo* fermentation liquid with time have a common path. The plot of changes reflected the degree of microbiota maturation in the indigo fermentation fluid. The stable state (phase 2) of an indigo fermentation liquor was shown to be a state in which the change of the microbial flora was extremely slow, as seen in Batch 1. Jaccard distance PCoA plots illustrated the differences between Batch 1 and Batches 2 and 3 in the transition from stable to terminal states, which is attributed to the initial heat treatment (once in Batch 1 and twice in Batches 2 and 3). Unweighted UniFrac distance PCoA plots illustrated the process of microbiota changes during the entire fermentation period regardless of the different microbiota. The pattern of changes in microbiota in Batch 3 differed slightly from those in the other batches. This finding is probably a reflection of the simpler microbiota composition in Batch 3 because of the absence of the facultative anaerobes *Bacillaceae* and *Pseudomonas* spp., both of which were present in Batches 1 and 2.

In Batches 1 and 2, the bacterial communities changed faster during days 0–28 and days 1–35 than after day 28 and day 35, respectively. This finding indicates that Batch 2 entered mature phase 2 on day 35, which was equivalent to that noted on day 342 in Batch 3 (Figure 4A). Therefore, for long-lasting fermentation in a stable ecosystem, it is necessary to enter mature phase 2 at a microbiota state equivalent to that on day 56 in Batch 1 (Figure 4B).

### Microbial Interaction Network

A relatively strong network was constructed on the basis of the interaction among the bacteria that survived the initial hot wood ash extract treatment (60°C, pH 11) in each batch. We considered that this state corresponds to phase 1. Initially appeared aerobic bacteria disappeared at a later time point in Batch 3 than in Batch 1; this finding was probably due to the fact that the relationship constructed from the bacteria initially present (mainly aerobic bacteria) in Batch 3 was stronger than that in Batch 1. This strong-relationship community was composed of oxygen-metabolizing bacteria that increased by the easy-to-use substrates present in *sukumo*. It was reflected in the network (Phase 1) constructed in Batch 3 with the absence of *Proteinivoraceae*.

**FIGURE 5:**
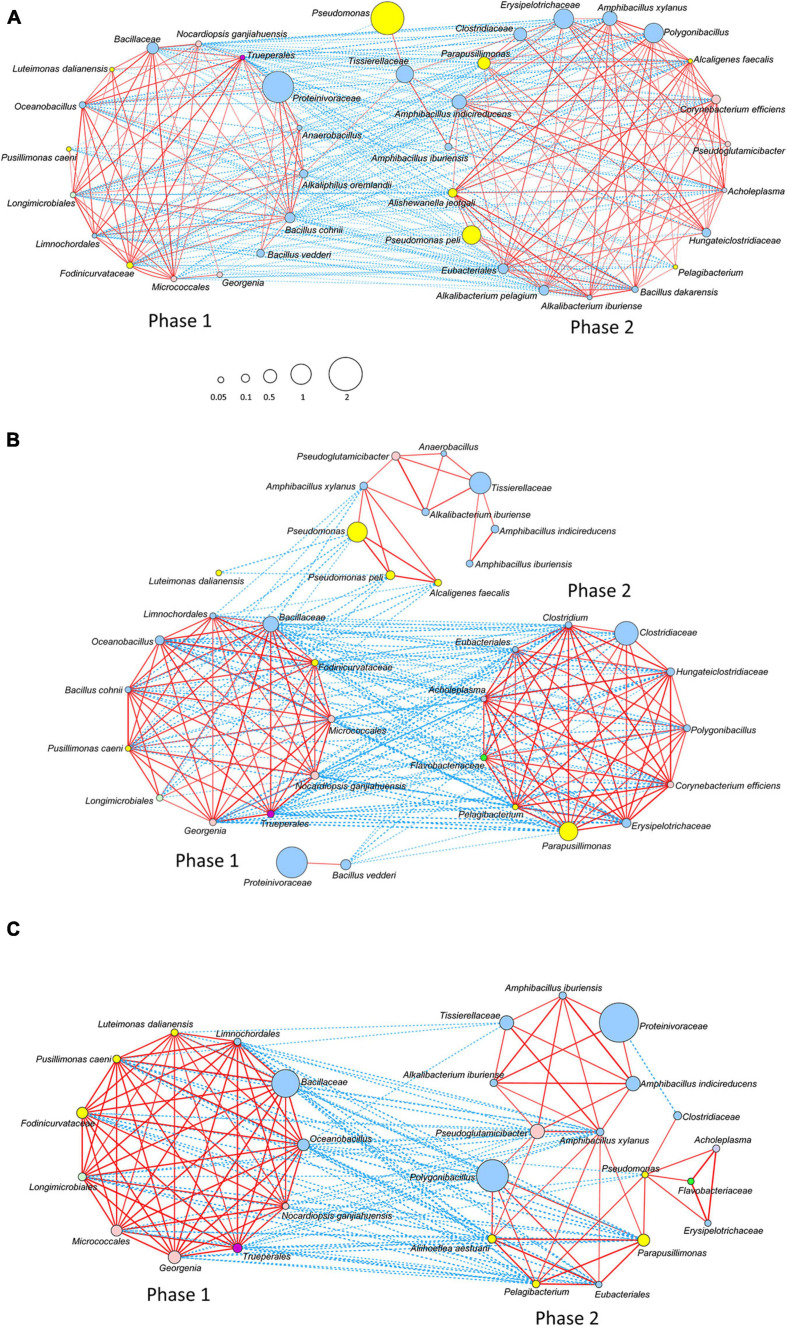
Networks of the bacterial community based on the relative content change trend analyzed with Spearman’s rank correlation coefficient [*rs* > 0.4, *p* < 0.05; **(A)**] Batch 1; **(B)** Batch 2; and **(C)** Batch 3. The red and blue lines represent positive and negative correlations, respectively. The thickness of the lines shows the strength of the relationship (0.4 < *rs* ≤ 1). Node size shows accumulated taxon abundances during the entire fermentation period. Classification at the phylum level is as follows: pale blue, *Firmicutes*; yellow, *Proteobacteria*; flesh color, *Actinobacteria*; bright green, *Bacteroidetes*; pale green, *Gemmatimonadetes* (*Longimicrobiales*); dark purple, *Deinococcus*-*Thermus* (*Trueperales*); and light purple, *Tenericutes* (*Acholeplasma*). The relative abundance is shown by the size of circle.

After the deterioration of the phase 1 network with a decrease in ORP followed by the primary and secondly substrates derived from *sukumo*, the phase 2 network was constructed on the basis of wheat bran as the substrate. There was a negative correlation between the initial phases and later phases, as indicated by the network change from phase 1 to phase 2 (Figures 5A–C). The ratio of the known indigo-reducing and plant-derived macromolecules (i.e., cellulose, xylan, and starch) utilizing *Amphibacillus*, *Alkalibacterium*, and *Polygonibacillus* was relatively low in Batch 2. In addition, *Amphibacillus* and *Alkalibacterium* were located in the weak network (Phase 2 in Figure 5B). This indicates that construction of the plants (*sukumo* or wheat bran)-derived macromolecule utilizing bacterial network was not successful. This is probably due to the fact that wheat bran was not supplied in Batch 2 (Table 1). These observations suggested that the transitional change in microbiota was not appropriate for supporting indigo reduction in Batch 2. In Batch 3, the network circle was relatively simple. The known indigo-reducing bacteria and obligate anaerobes were located in the network circle, and their relative ratios were high. The transitional changes in microbiota were observed to be clearly separated in phase 2 through transitional changes in the microbial network (Figure 5C). In Batch 3, a transitional change seemed to occur from early phase 2 and late phase 2 (Figure 5C). However, the contribution of the known indigo-reducing bacteria to the main network circle was decreased, and *Parapusillimonas*, *Erysipelotrichaceae* and *Clostridiaceae* were dominant in late phase 2.

### Potential Metabolic Differences Between Phase 1 and Phase 2

Analysis of the predicted metabolic functions potentially expressed by the microbial communities showed differences between phase 1 and phase 2 in Batch 1 samples (Supplementary Figure 4). Many items indicated that separate events occurred before and after the day 28 (data not shown). Therefore, the difference in the average ratio between phase 1 (days 0–28) and phase 2 (days 28–364) was estimated. There were many profiles representing changes in the relative abundance of amino acid metabolism and fatty acid degradation, as well as carbohydrate metabolism and glycan degradation, in phase 1 and phase 2, respectively, which indicated the differences in major nutrients for bacteria between phase 1 and phase 2. Relative abundances of riboflavin and lipoic acid metabolism in phase 1 was observed. This suggests that the basis of electron transfer systems related to indigo reduction was established in phase 1. The abundance in oxidative phosphorylation in phase 1 was mostly attributed to the oxygen metabolism after the initiation of fermentation. It is known that the phosphotransferase system is involved in transporting many sugars to bacteria, including glucose, mannose, fructose, and cellobiose. This might be related to the relative abundance of carbohydrate metabolism in phase 2. The abundance of biofilm formation in phase 2 suggested the development of biofilm formation on the debris derived from *sukumo*.

## Discussion

In the current study, to elucidate the mechanism underlying the sustainability of indigo reduction, three batches of indigo fermentation fluids with different indigo-reduction durations and different maintenance procedures were evaluated by analyzing changes in the microbiota, by heat map clustering, by microbial interaction network analyses, and by functional prediction of the metagenome. The results showed that a transitional change from phase 1 (initial phase) to phase 2 (mature phase), in accordance with changes in substrates of major bacteria, is important for subsequently entering a long-lived indigo-reducing state. The microbiota in phase 1 consumed substrates including intrinsic substrates in *sukumo* and weakened or dead bacterial cells derived from early events (heat and alkaline treatment and reduced ORP) within approximately the first 1 month. After the exhaustion of the substrates by phase 1 bacteria, the microbiota including known indigo-reducing bacteria that can decompose macromolecules in wheat bran (i.e., *Amphibacillus* and *Polygonibacillus*; Hirota et al., 2013a, b, 2016; Nishita et al., 2017) appeared at a high rate in phase 2.

It was reported that the selection of microorganisms for the initial treatment with hot wood ash extract is important for the selection of necessary microorganisms, which consume oxygen in the early fermentation period (Tu et al., 2019a). In addition, entering stable alkaline anaerobic conditions dominated by obligately anaerobic *Anaerobranca* is important for the maintenance of an indigo reduced state of fermentation for a long duration (Tu et al., 2019b). Thus, the microbiota in the original material, *sukumo*, suffered twice from successive environmental pressures (hot wood ash extract and the disappearance of oxygen) at the beginning of the fermentation. However, the availability of substrates for the surviving microorganisms was exhausted at approximately 1 month after the beginning of fermentation. The earlier phase, Phase 1, could be strongly influenced by the main material used to produce the fermentation fluid, *sukumo*. Each corresponding intrinsic bacterial community resulted from the preparation procedures. It can be assumed that bacterial substrates, such as degraded components of *P*. *tinctorium* leaves and dead bacterial cells derived from the composting process, are present in *sukumo*. In addition, damaged bacterial cells by hot wood ash extract (60–70°C, pH 10.8–11.5) may be produced at the beginning of the fermentation. In addition, dead bacterial cells resulting from the initial treatment may be candidate substrates for obligate anaerobes (i.e., *Proteinivoraceae* and *Tissierellaceae*) as the taxa related to obligate anaerobes are known as sludge decomposers (Maspolim et al., 2015; Wang et al., 2019). The dead or weakened bacterial cell components may also be derived from transitional changes in the predominant bacteria, especially in the decrease in ORP at the early fermentation stage. The decomposition of dead bacterial cells would be promoted chemically under high alkaline conditions (Wang et al., 2019). In the later phase, after the amount of substrates decreased, the second major substrates for the bacteria were wheat bran, which was added to the fermentation fluids at days 22 and 35 in Batches 1 and 3, respectively. Further, the debris derived from *sukumo* remaining from the earlier phase of the fermentation can also be considered a bacterial substrate in phase 2. Both wheat bran and the debris derived from *sukumo* contain insoluble macromolecules (i.e., cellulose, xylan, and starch; Xie et al., 2008; Rémond et al., 2010) that are normally not easily decomposed by ordinary bacteria. The difficulty in decomposing insoluble macromolecules in wheat bran may lead to slow transitional changes in microbiota.

*Sukumo* was pre-treated with hot (60–70°C) ash extract (ca. pH 11) during the preparation procedure, which led to the selection of *Bacillaceae* and *Actinobacteria*. Because of oxygen metabolism by the aerobes and facultative anaerobes, the predominant bacterial population changed from the originally present bacteria in days 0–1 to obligate anaerobes (mainly *Proteinivoraceae*). Another study showed that a substantial change occurs in the microbiota during the early stage of fermentation, which is characterized by the appearance of *Bacillaceae* and subsequently by *Anaerobranca* sp. (93.1% similarity to *Anaerobranca gottschalkii* and this species belongs to *Proteinivoraceae*; Tu et al., 2019b). A previous research has shown that an initial drop in ORP is not directly linked to indigo reduction (Aino et al., 2010). In the current study, data from three different batches showed that indigo reduction occurs when the ratio is reversed from *Bacillaceae* to *Proteinivoraceae* as the dominant group in *sukumo* fermentation fluids and that the change is connected to the initiation of indigo reduction. These findings suggested that both reduction of the initially dominant bacteria and the appearance of the absolute anaerobic *Proteinivoraceae* are necessary for initiation of indigo reduction. Actually, the sudden increase in *Proteinivoraceae* always occurs after a decrease in the initially existing bacteria derived from *sukumo*. This suggests that initiation of indigo reduction may be related to a decrease in the initially exiting bacteria derived from *sukumo*. There is a possibility that a decrease in oxygen-metabolizing or facultative anaerobic bacteria is associated with a release of electron mediators such as flavins or quinones from the dead or damaged cells. The extracellular electron transfer mechanism mediated by flavin has been reported (Light et al., 2018, 2019).

The predominance of *Proteinivoraceae* gradually decreased after 14 days in Batch 1. This may indicate a decrease in substrates for this taxon, which were abundant when this bacterial species was prioritized. In addition to *Proteinivoraceae*, obligate anaerobic *Tissierellaceae* was observed in succession. *Proteinivoraceae* (formerly *Anaerobrancaceae*) hydrolyzes sludge protein to short-chain fatty acids under anaerobic high pH conditions (Wang et al., 2019). Moreover, several studies have reported that *Proteinivorax*, a member of *Proteinivoraceae*, produces proteases (Lavrentyeva et al., 2019). Furthermore, *Proteinivorax* bacteria lyse dead and/or damaged cells of *Halomonas campisalis* (Boltyanskaya and Kevbrin, 2016). *Tissierella* produces volatile fatty acids during anaerobic sludge fermentation under alkaline conditions (Maspolim et al., 2015; Huang et al., 2018). These findings suggest that these obligate anaerobes have similar functions in sustaining the indigo-reducing system.

The predominating of *Proteinivoraceae* in phase 1 was tentatively succeeded by *Clostridiaceae* (day 28 in Batch 1), followed by *Amphibacillus* (day 56). PCoA showed that after day 28, the rate of transitional changes in the microbiota became low, indicating that the bacterial flora may have entered a stable phase because of the influence of substrates derived from *sukumo*, and that the initial events (i.e., heat treatment and decrease in ORP) in the fermentation decreased within approximately 1 month. The finding suggested that the different bacterial species showed parallel competition for niches. The dominating obligate anaerobes decreased from phase 1 (initial stage) to in phase 2 (mature stage) but maintained certain ratios (10–15%). After *Clostridiaceae* decreased, *Amphibacillus* initially dominated, followed by *Alkalibacterium* and then *Polygonibacillus*. All these genera have been reported to include indigo-reducing taxa and are often found in various indigo fermentation fluids (Aino et al., 2018). The increase in the ratios of *Am*. *indicireducens* and *Al*. *iburiense* may be due to the addition of wheat bran because these microorganisms can degrade cellulose, xylan and starch, which are components of wheat bran (Yumoto et al., 2004, 2008; Nakajima et al., 2005; Hirota et al., 2013a, b). *Polygonibacillus indicireducens* is able to decompose starch or cellulose (Hirota et al., 2016, 2020). The bacterial flora sustained by wheat bran may utilize the remaining substrates in *sukumo*. It can be considered that multiple functional redundancies, including those of several known indigo-reducing bacteria, were established during this phase to support the indigo-reducing state.

The microbiota was relatively stable during phase 2. However, because of the development of neutralophilic microenvironments in the long term, the neutralophilic bacterial ratio and bacterial diversity gradually increased. Our findings indicated that the increase in diversity led to dilution of the functional network to sustain indigo-reduction mechanisms. In addition, the staining intensity results suggested that a decrease in the ratio of *Proteinivoraceae* and an increase in the ratio of the *P. granuli*-like taxon may unfavorable for sustaining indigo reduction. Our results also suggested that a decrease in the ratio of indispensable taxa, an increase in taxa unfavorable for indigo reduction, exhaustion of substrates sustaining phase 2, or the accumulation of metabolic by-products would have led to the transition from a network favorable for maintaining indigo reduction to the next stage. We believe that the transition from the favorable network to the next stage would have altered the indigo-reduction state.

Among the three batches, only Batch 2 was short-lived. The disadvantage for maintaining the indigo-reducing state in Batch 2 was the low ratio of known indigo-reducing bacteria. This is probably related to the lack of wheat bran in Bach 2, because mostly known indigo-reducing bacteria utilize substrates in wheat bran. The maximum abundance of *Am*. *indicireducens* was approximately 6% in Batch 2, but was approximately 26.1% and 15.7% in Batches 1 and 3, respectively. This ratio is consistent with the amounts of introduced wheat bran. Since the substrate in the stable period is considered to be wheat bran, it is considered that Batch 2 to which wheat bran was not added did not successfully transition to phase 2. This was also revealed in the network with two networks divided with no positive correlation (Figure 5B). If the number of microorganisms that can decompose water-insoluble macromolecules such as cellulose in *sukumo* increased by the time of transition to Phase 2, a stable period could have been formed. Another disadvantage in Batch 2 causing the low ratio of indigo-reducing bacteria was probably the high pH during the early fermentation stage. The pH of the fermentation fluid remained at approximately 11.2–11.5 for the first 10 days after the initiation of fermentation (Supplementary Figure 1). A high pH is thought to effectively eliminate species unfavorable for indigo fermentation. However, a long period of exposure to high pH (>11) is responsible for damage to bacterial beneficial for indigo reduction. Therefore, regulation of the initial pH is very important for the generation of favorable long-lasting microbiota. On the whole, bacteria that decompose plant macromolecules in phase 2 weakened in the early stage of the fermentation, and deficiency of substrates paramount for phase 2 led to the failure of the transition from phase 1 to phase 2. It is considered that these shortages resulted in the short-lived indigo-reducing state in Batch 2. Since the reduction state of the fermented fluid can usually be evaluated by the staining status, the transition of the bacterial community has quietly occurred 3 weeks before (i.e., day 28) the significant change in the staining status (i.e., day 49 of Batch 2). This study found that adding wheat bran within the first month (i.e., between day 21 to 28) of fermentation will help the smooth transition of the bacterial community.

The purpose of this research was to clarify the mechanisms of underlying the maintenance of an indigo reducing state in the indigo fermented liquid under an environment wherein the indigo fermented liquid is naturally fermented, and contamination can occur frequently. In this research, we focused on the transitional changes of the microbial flora responsible for the indigo reduction and aimed to achieve this purpose. However, the results obtained were that the microbial community supporting the reduction of indigo brought about by the raw material, *sukumo*, was disrupted by the reduction of the major nutrient sources of the microbial community that first appeared, and the subsequently added wheat bran was the main nutrient. This occurred to replace the microbial community that initially appeared. This means that there is functional overlap in the background microbial flora (Luan et al., 2020; Tannock, 2021; Zhao et al., 2021), and the major substrates can produce a network via another microbial community. It was speculated that the slow transition of the microbial flora occurred due to the substrates, which slowly decomposed by microbial community in phase 2, but the real resiliency mechanism in detail of the last microbial network formed has not been clarified. The sustainable ecosystem in the later phase should be explained on the basis of the functions of constituent microorganisms and the material cycle in fermentation in detail.

In the future, the more detailed relationship between existing substrates and microorganisms should be clarified. We would like to understand the changes in the material cycle related to the function of the bacterial constituents in the fermentation liquid, which was maintained for a long period, in more detail. This knowledge will lead to enhancements in the sustainability of indigo reduction fermentation systems. It is considered that the obtained knowledge could be applied to sustainable ecosystems comprising utilization of starch, cellulose, and xylan. For example, it could be applied to highly sustainable microbial respiratory fuel cells under alkaline environments (Hobbie et al., 2012; Fuller et al., 2014; Wang et al., 2018).

## Data Availability Statement

The datasets presented in this study can be found in online repositories. The names of the repository/repositories and accession number(s) can be found below: https://www.ddbj.nig.ac.jp/, DRA011373.

## Author Contributions

ZT and IY: conceived and designed the experiments and wrote the manuscript. ZT and HL: performed the experiments. ZT, TN, and IY: analyzed the data. All authors contributed to the article and approved the submitted version.

## Conflict of Interest

The authors declare that the research was conducted in the absence of any commercial or financial relationships that could be construed as a potential conflict of interest.

## Publisher’s Note

All claims expressed in this article are solely those of the authors and do not necessarily represent those of their affiliated organizations, or those of the publisher, the editors and the reviewers. Any product that may be evaluated in this article, or claim that may be made by its manufacturer, is not guaranteed or endorsed by the publisher.

## Footnotes

1 https://blast.ncbi.nlm.nih.gov/Blast.cgi
