# Supplementary material for: The Mechanism Underlying of Long-Term Stable Indigo Reduction State in Indigo Fermentation Using Sukumo (Composted Polygonum tinctorium Leaves)
Source: Front Microbiol. 2021 Jul 23;12:698674. doi: 10.3389/fmicb.2021.698674 (PMC8342947; doi:10.3389/fmicb.2021.698674)
Supplement: Supplementary file 1 [file Data_Sheet_1.docx]

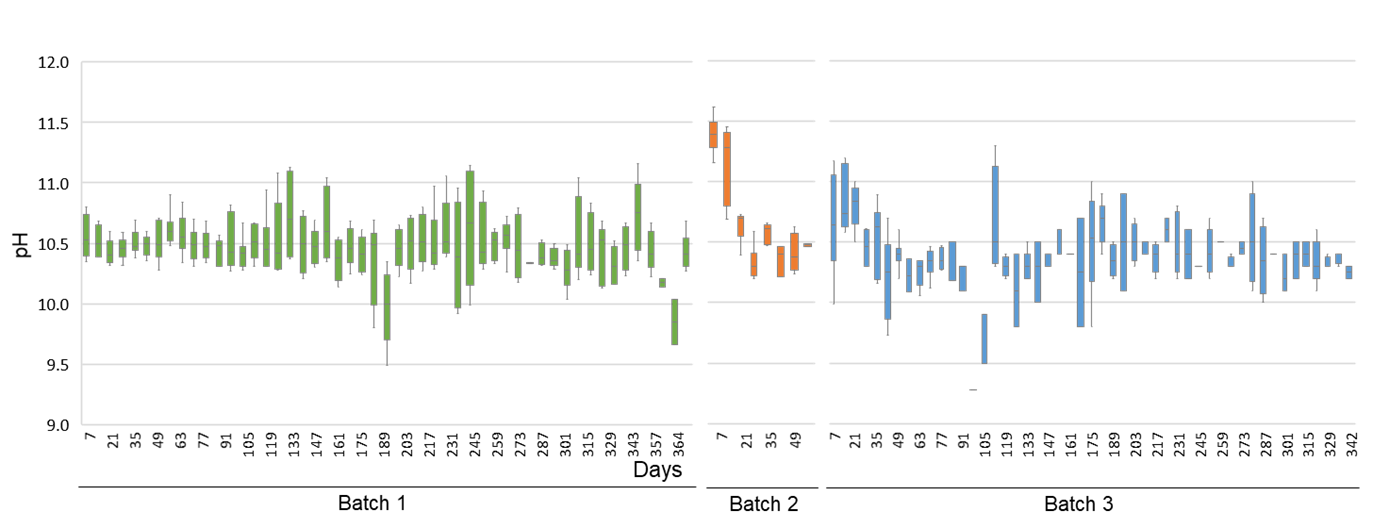


**Fig. S1**  Ranges of weekly pH changes of Batch 1–3 fermentation fluids during each incubation period.


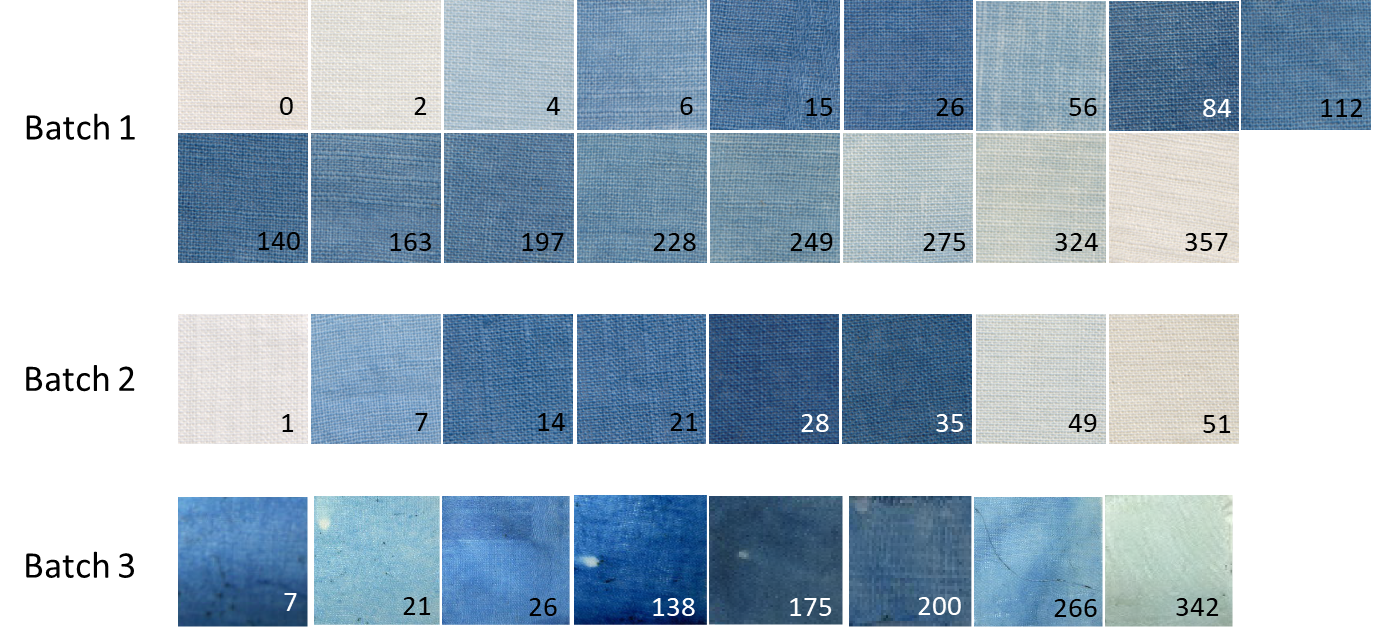


**Fig. S2** The blue color depth of dyed cotton cloth shows the indigo-reduction sate. The numbers are fermentation days for each batch. Dyeing textiles scanned in the same condition in the samples from Batches 1 and 2, while those from Batch 3 were photographed at each time point independently.


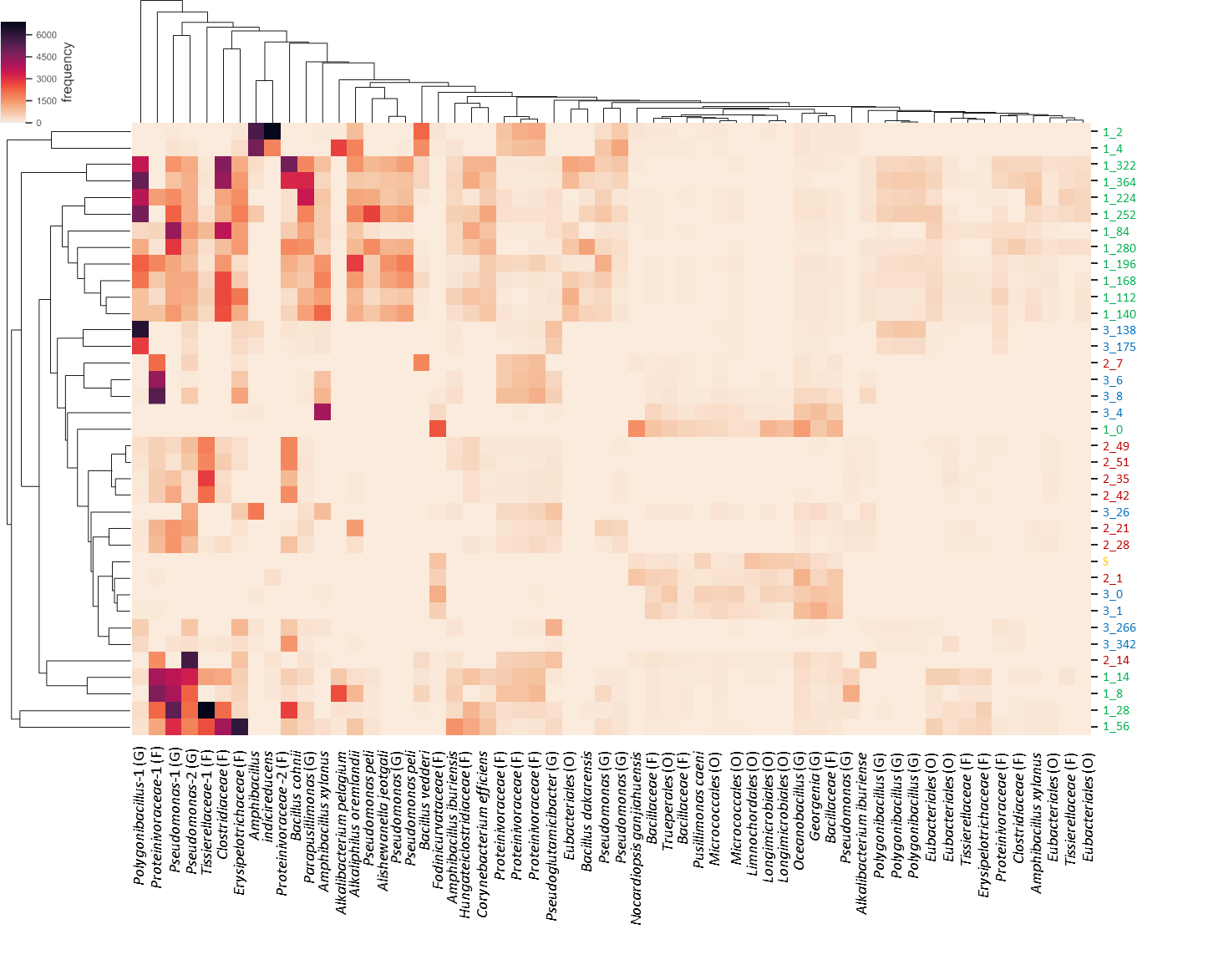


**Fig. S3** Clustering heat map based on the relative abundance of operational taxonomic units (OTUs) detected in the indigo fermentation Batches 1–3. The resulting out sequences corresponding OTUs were searched in the BLAST database. The classification hierarchy described in brackets depends on database similarity as follows: O, order level (<90% similarity); F, family level (≥90, <95%) and G, genus level (≥95, <98.6%). Batches 1, 2, and 3 are green, red and blue letters, respectively. They show the batch number and incubation period combined. S: *sukumo*

**Fig. S4.** Difference in relative abundances between phase 1 and phase 2 in Batch 1 based on KEGG categories predicted by PICRUSt2 (Level 2 and KEGG pathway mapping). The comparison was made based on the average difference in abundance between phase 1 and phase 2 for each item. The KEGG pathway entry number is shown brackets in the KEGG pathway mapping column.
